# Interrelations of resilience factors and their incremental impact for mental health: insights from network modeling using a prospective study across seven timepoints

**DOI:** 10.1038/s41398-023-02603-2

**Published:** 2023-10-23

**Authors:** Sarah K. Schäfer, Jessica Fritz, M. Roxanne Sopp, Angela M. Kunzler, Lisa von Boros, Oliver Tüscher, Anja S. Göritz, Klaus Lieb, Tanja Michael

**Affiliations:** 1 Leibniz Institute for Resilience Research, Mainz, Germany; 2 Department of Clinical Psychology and Psychotherapy, Saarland University, Saarbrücken, Germany; 3 Department of Clinical Psychology, Psychotherapy and Psychodiagnostics, Technische Universität Braunschweig, Braunschweig, Germany; 4 Department of Psychiatry, University of Cambridge, Cambridge, United Kingdom; 5 Department of Clinical Psychology, Philipps-University Marburg, Marburg, Germany; 6 Institute for Evidence in Medicine, Medical Center–University of Freiburg, Faculty of Medicine, University of Freiburg, Freiburg, Germany; 7 Department of Psychiatry and Psychotherapy, University Medical Center of Johannes Gutenberg University, Mainz, Germany; 8 Institute for Molecular Biology, Mainz, Germany; 9 Behavioral Health Technology, Augsburg University, Augsburg, Germany

**Keywords:** Pathogenesis, Diseases

## Abstract

Resilience can be viewed as trajectory of stable good mental health or the quick recovery of mental health during or after stressor exposure. Resilience factors (RFs) are psychological resources that buffer the potentially negative effects of stress on mental health. A problem of resilience research is the large number of conceptually overlapping RFs complicating their understanding. The current study sheds light on the interrelations of RFs in the face of the COVID-19 pandemic as a use case for major disruptions. The non-preregistered prospective study assessed a sample of 1275 German-speaking people from February 2020 to March 2021 at seven timepoints. We measured coping, hardiness, control beliefs, optimism, self-efficacy, sense of coherence (SOC), sense of mastery, social support and dispositional resilience as RFs in February 2020, and mental health (i.e., psychopathological symptoms, COVID-19-related rumination, stress-related growth) at all timepoints. Analyses used partial correlation network models and latent growth mixture modeling (LGMM). Pre-pandemic RFs were strongly interrelated, with SOC being the most central node. The strongest associations emerged between coping using emotional support and social support, SOC and sense of mastery, and dispositional resilience and self-efficacy. SOC and active coping were negatively linked. When we examined RFs as predictors of mental health trajectories, SOC was the strongest predictor of psychopathological symptoms and rumination, while trajectories of stress-related growth were predicted by optimism. Subsequent network analyses, including individual intercepts and slopes from LGMM, showed that RFs had small to moderate associations with intercepts but were unrelated to slopes. Our findings provide evidence for SOC playing an important role in mental distress and suggest further examining SOC’s incremental validity. However, our results also propose that RFs might be more important for stable levels of mental health than for adaptation processes over time. The differential associations for negative and positive outcomes support the use of multidimensional outcomes in resilience research.

## Introduction

Resilience can be viewed as stable good mental health or fast recovery of mental health during or after stressor exposure [1, 2]. According to this outcome-based approach, resilient outcomes are partially determined by a variety of resilience factors (RFs) that are supposed to buffer the potentially negative effects of stressor exposure on mental health mediated via a smaller number of resilience mechanisms (e.g., positive appraisal style [1], regulatory flexibility [3]). RFs may comprise dispositional variables and resilience-promoting traits (e.g., optimism [4]), beliefs (e.g., self-efficacy [5], control beliefs [6]), and coping strategies [7] (see Table 1). Over the past decades, the number of potential RFs has increased enormously, resulting in conceptual overlaps and strong empirical interrelations [8].

**Table 1:** List of studied resilience factors.

Resilience factor	Definition
Active coping (e.g., problem-solving)	Coping as a set of intentional, goal-directed efforts to minimize physical, psychological and social harm of stressors [83]; active coping as utilization of psychological and behavioral efforts that use own resources to handle a stressor [100].
Cognitive emotion regulation (e.g., positive reframing)	Conscious thoughts by means of which individuals regulate their emotions in response to stressors [26]. Positive reframing, as a type of reappraisal, describes thinking about negative or challenging situations in a positive way (e.g., thinking about the benefits or upsides of a negative event) [101].
Humor	Humor represents the capacity to perceive or express the amusing aspects of situations [102].
Hardiness	Hardiness is an ability to handle unexpected changes (challenges) with ease, combined with a sense of meaning in daily life (commitment) and personal control (control) [92].
Locus of control/control beliefs	Locus of control represents the degree to which people believe that they have control over outcomes in their lives [6]. A strong internal locus of control reflects the belief that outcomes are primarily a result of own action, an external locus of control is associated with viewing external factors as primary causes of outcomes.
Optimism	Optimism reflects the extent to which people hold generalized favorable expectancies for the future [4].
Religiosity or spirituality	Religiosity and spirituality describe any feeling, thought, and behavior that arises from the search for the ‘sacred’. Religiosity also includes group or social practices, while spirituality refers to personal beliefs and experiences [103].
Self-efficacy	Self-efficacy describes an individual’s subjective perception of his or her capability to perform a specific behavior or to achieve something [5].
Sense of coherence	Sense of coherence is the key component of the salutogenesis concept [14]. Individuals with high levels of sense of coherence perceive their lives as comprehensible, manageable and meaningful [80]. Comprehensibility describes the perception of the environment as predictable, structured, and explicable. Manageability refers to the belief that available internal and external resources are sufficient to meet situational demands, and meaningfulness describes the belief that challenges are worthy of engagement and coping.
Sense of mastery	Sense of mastery describes a person’s belief that they are able to control important circumstances in their life [104].
(Perceived) social support	Perceived social support describes the network of social resources perceived by an individual [105].
Dispositional resilience	Dispositional resilience is a personality trait that helps individuals to cope with adversity and achieve successful adjustment and development in the face of stressors [106]. The concept is closely related to hardiness [92] .

The selection of resilience factors is based on an updated summary of resilience factors presented in Kunzler et al. [11]. and Schäfer et al. [26]. Other concepts that are discussed as resilience factors are hope (closely related to optimism), meaning and purpose in life (closely related to the meaningfulness component of sense of coherence), positive emotions/affect and self-esteem, with the latter two being investigated as resilience factor, resilience mechanism and resilience outcome [107, 108]. Recently introduced concepts like regulatory flexibility [93] were not included as they have not been examined in systematic reviews on resilience factors yet.

For instance, control beliefs [6] are closely related to self-efficacy [5]. On a conceptual level, it may be argued that one can perceive a situation as potentially controllable by oneself (internal locus of control) but does not believe in one’s own capacities to manage the situation (low self-efficacy belief). However, it is unlikely that a person with strong situation-specific self-efficacy beliefs views a situation as externally controlled (external locus of control). This example is just one of many that underscores how poorly some RFs can be differentiated. The missing conceptual clarity hampers the planning of studies on resilience, as they often include a large number of similar and time-consuming RF questionnaires [9], and is a challenge for resilience interventions. Even though resilience is nowadays often understood as an outcome, the mediating mechanisms linking RFs to resilient outcomes are still insufficiently understood [1, 2]. Therefore, resilience trainings primarily target RFs, with the outcomes of intervention studies being operationalized as changes in RFs (and mental health) [10, 11]. Thus, reducing the large number of potential intervention targets in terms of RFs could advance the development and evaluation of resilience interventions.

Research on the overlap and incremental validity of single RFs is not new [12], and many studies aimed at selecting the most important RFs [13]. Most of these studies were solely based on cross-sectional data, employed regression models and many studies only examined a limited set of RFs. One of the RFs that has been shown to have incremental validity above other factors is sense of coherence (SOC), that is, a global orientation that life is comprehensible, manageable, and meaningful [14, 15]. In previous studies, SOC was found to show incremental validity beyond optimism, self-efficacy, locus of control, coping, and dispositional resilience [16–21], leading to the theoretical claim that SOC may combine relevant aspects of other RFs [12]. Although there is evidence for SOC showing incremental validity above other factors, none of those studies used large-scale longitudinal data or examined a larger set of well-established RFs.

Here we set out to use network analyses to shed light on overlapping and most likely interrelated RFs [22]. Network analysis allows to estimate and visualize associations between variables without providing information on the underlying dimensional structure, as is mandatory for other approaches [23]. First studies have employed network models in the field of resilience research to investigate how RFs relate to each other [24, 25]. A study using a network approach on RFs [24] compared a network of RFs for adolescents with and without exposure to childhood adversity, finding that the interrelatedness of RFs was higher in the non-exposed group and that the network model in the exposed group comprised more negative relations. This suggests that various RFs rather hamper than support each other. A similar approach was chosen by Thoma et al. [25], who compared network models of RFs and stress-related risk factors for older adults with and without early-life adversity. They found a larger number of relevant associations in the group without early-life adversity, while the impact of current stress was stronger in the stress-exposed group.

Being the hitherto largest and most disruptive global stressor of the 21st century, the COVID-19 pandemic is an important use case for resilience research [26]. Stress caused by COVID-19 not only results from the fear of the virus but also from the impact of containment measures [27]. As stress is one of the leading causes of the onset and persistence of mental disorders [28], it is not surprising that the COVID-19 pandemic had serious negative mental health consequences for substantial parts of the population [29]. In line with this notion, meta-analyses found increased symptom distress [26, 30] and elevated rates of depressive and anxiety disorders in the general population [29].

To our knowledge, only a small number of studies employed a network model approach to RFs in the context of the COVID-19 pandemic [31–33]. However, only one study on young adults examined a broad set of RFs and used prospective data [33]. Independent from the use of network models, there is little knowledge on RFs, their interrelations, and predictive^1^ values for mental health during the pandemic. Existing evidence remains mostly limited to the first wave of the pandemic until the summer of 2020 [26]. Most research has been conducted on social support, which may be caused by the potential harm due to social isolation during the pandemic [34]. While some studies found higher levels of social support to be associated with better mental health [35–37], other studies examining a broader set of RFs did not find a significant association between social support and mental health [13, 38]. Similarly, with respect to cognitive emotion regulation, findings were mixed for different strategies [13, 39] and also for single strategies in different studies [13, 35]. To our knowledge, only a small share of longitudinal studies compared the predictive value of a broader set of RFs [13, 38, 40], with many only investigating different aspects of a single RF and none of the studies focusing on the relationship between RFs or a broader range of stress-related outcomes including positive aspects of mental health. Thus, a prospective study examining the associations of a broad set of RFs and their unique predictive value for trajectories of mental health is missing.

Using the COVID-19 pandemic as a use case, the current study aimed at: (1) examining the network of pre-pandemic RFs; (2) studying those RFs as predictors of mental responses to the pandemic (i.e., trajectories of psychopathological symptoms, COVID-19-related rumination, stress-related growth^2^); (3) investigating the links between RFs and characteristics of individual responses to the pandemic (i.e., intercepts and slopes of individual trajectories). The latter analyses aimed at examining whether RFs are related to (probably rather stable) mean-level differences in mental health and distress and/or differences in dynamics over time. Building on previous studies showing the incremental validity of SOC beyond other RFs [12, 16–21], we assumed SOC to be a strong component of the pre-pandemic RF network and to predict trajectories of psychopathological symptoms and rumination over time. We had no hypothesis for stress-related growth. Associations with intercepts and slopes were examined on an exploratory basis.

## Methods

### Study design and sample recruitment

The current study derived from a non-preregistered cross-sectional study on RFs conducted in February 2020. In March 2020, the study team decided to widen the scope of the project and investigate the association between SOC and psychopathological symptoms during the COVID-19 pandemic [42, 43]. Six follow-up assessments took place until March 2021. Assessment points were chosen to capture critical points of the pandemic (see Supplementary Material SM1). For sample recruitment, we used the WiSoPanel [44] holding *N* = 14,369 German-speaking adults who live in Germany, Austria, Switzerland, or border regions in neighboring countries. The panel holds socioeconomically diverse individuals with heterogeneous demographic backgrounds. All respondents registered for the WiSoPanel were eligible for inclusion. There were no specific in- and exclusion criteria for this study. Data was collected online via SoSci Survey [45]. The study was conducted in accordance with the ethical standards of the ethics committee of Saarland University but was exempted from approval. All respondents provided informed consent. For the present analyses, we used data from 1275 respondents (63.5% of the baseline sample) who completed at least two assessments of mental health outcomes.

### Measures

Resilience factors were assessed at the pre-pandemic baseline in February 2020, mental health outcomes were collected longitudinally until March 2021 (see Supplementary Fig. S1 for a detailed data collection plan).

#### Mental health outcomes

##### COVID-19-related rumination

COVID-19-related rumination was assessed starting in March 2020 using a modified version of the Perseverative Thinking Questionnaire [46]. The 15-item instrument assesses core characteristics of rumination and was mildly adjusted to assess COVID-19-related rumination [43] (see Supplementary Material SM2). Each item was rated on a 5-point scale, and higher scores indicate more intense rumination. Internal consistency was excellent at all assessments (Cronbach’s alpha [α] = 0.97; McDonald’s omega [ω] = 0.97).

##### Psychopathological symptoms

Psychopathological symptoms were measured using the Brief Symptom Inventory-18 [47], a short version of the Brief Symptom Checklist. The 18-item scale is a measure of general psychopathological symptom burden. Each item is rated on a 5-point scale, with higher scores indicating more severe symptoms. The internal consistency was excellent (range across assessments: α/ω = 0.94–0.95).

##### Stress-related growth

Stress-related growth was assessed starting in April 2020 using an adapted version of the Posttraumatic Growth Inventory [48]. The 21-item scale was modified to assess COVID-19-related growth (see Supplementary Material SM3). Each item was rated on a 5-point scale. Higher scores indicate higher levels of growth. The internal consistency was excellent at all assessments (α/ω = 0.96).

#### Resilience factors

Table 1 presents brief definitions of all RFs examined in this study.

##### Adaptive coping

The Brief COPE Inventory [49] was used to assess coping strategies. The 28-item instrument measures 14 coping strategies, each with two items, based on a 4-point scale. For the current analyses, we used the Brief COPE subscales showing negative, at least marginally significant, cross-sectional associations with psychopathological symptoms at baseline: active coping (AcC; *r* = −0.05, *p* = 0.017), emotional support (EmS; *r* = −0.04, *p* = 0.065), and positive reframing (PoR; *r* = −0.06, *p* = 0.013). These subscales showed acceptable to good internal consistencies (active coping: α/ω = 0.71; emotional support: α/ω = 0.83; positive reframing: α/ω = 0.76).

##### Hardiness (Hard)

Hardiness was assessed using a German translation of the Dispositional Resilience Scale [50, 51]. The 15-item scale assessed hardiness using a 4-point scale, with higher scores indicating higher levels of hardiness. The scale demonstrated good internal consistency (α/ω = 0.84).

##### Internal locus of control (LoIn)

Internal and external locus of control were assessed using the Brief Scale for the Assessment of Internal and External Control Beliefs [52]. The instrument consists of two subscales measuring internal and external control, each comprising two items. All items are rated on a 5-point scale. For the current study, we used the internal locus of control subscale with good internal consistency (α/ω = 0.80).

##### Optimism (Opt)

Dispositional optimism was assessed using the Scale for Optimism-Pessimism 2 [53]. The 2-item scale assesses optimism and pessimism on a 7-point scale. For the current study, we used the optimism item. Higher scores indicate higher levels of optimism.

##### Self-efficacy (SE)

Generalized self-efficacy was assessed using the General Self-Efficacy Short Scale [54]. The 3-item scale assesses self-efficacy on a 5-point scale, and higher scores indicate stronger self-efficacy. The internal consistency was excellent (α/ω = 0.91).

##### Sense of coherence (SOC)

SOC was assessed using the 9-item German short version [55] of the Orientation to Life Questionnaire [56]. The measure uses a bipolar 7-point scale with a verbal anchor at each pole. Higher scores indicate higher levels of SOC. The scale showed excellent internal consistency (α/ω = 0.90).

##### Sense of mastery (SOM)

Sense of mastery was assessed using a German version of the Pearlin Mastery Scale [57]. The 4-item instrument uses a 4-point scale. Higher scores reflect a stronger sense of mastery. The scale demonstrated good internal consistency (α/ω = 0.89).

##### Social support (SOS)

Perceived social support was assessed using the Brief Form of the Perceived Social Support Questionnaire [58]. The 6-item instrument uses a 5-point scale, and higher scores indicate higher levels of social support. The scale showed excellent internal consistency (α = 0.92; ω = 0.91).

##### Dispositional resilience (Res)

The Resilience Scale 13 [59] was used to assess dispositional resilience (i.e., resilience as a personality trait). The scale is a German short version of the Wagnild and Young Resilience Scale [60]. Dispositional resilience is assessed using 13 items rated on a 7-point scale, and higher scores indicate higher levels of dispositional resilience. The scale showed excellent internal consistency (α/ω = 0.94).

### Statistical analyses

All analyses were performed using R version 4.4.2 [61] and Mplus version 8.10 [62].

#### Missing data

The *Rbtest* package [63] was used to test for types of missing data (i.e., missing completely at random, missing at random). For data missing completely at random or missing at random, we performed full information maximum likelihood (FIML) estimations [64].

#### Trajectory modeling

For the study of mental responses to the pandemic, we employed the most common approach used in resilience research, that is, latent growth mixture modeling [65, 66] (LGMM). LGMM is a method to identify multiple unobserved sub-populations from an overall non-normal distribution describing different patterns of change in those sub-populations [67]. We used LGMM to examine trajectories of psychopathological symptoms, COVID-19-related rumination, and stress-related growth. As the number of assessments was different between outcome types and we expected different responses per outcome type, we ran separate models for each outcome. Models were estimated using FIML and robust standard errors (MLR) to account for non-normally distributed data. First, we identified the best-fitting class solution for each outcome. In line with the methodological criticism raised for highly constrained models [68, 69], all models allowed for within-class variation of intercepts and slopes. For each mental health outcome, we examined linear and quadratic slopes. In line with recent recommendations [70], models with increasing numbers of classes were compared by means of Akaike information criteria (AIC) and sample-size-adjusted BIC (SSBIC). The significance of fit differences was indicated by the bootstrapped likelihood ratio test (BLRT), with a significant test indicating the usefulness of including another class. For reasons of insufficient reproducibility and stability, solutions with very small classes (≤5%) were excluded. The choice of best-fitting solutions was based on fit indices and theoretical coherence. Moreover, we report on Entropy (range: 0–1, higher scores indicate better class separation), which has not been used for model selection [71]. For the best-fitting models, individual intercepts as well as linear and, if applicable, quadric slopes accounting for the most likely class membership were saved as factor scores for later network analyses.

Subsequently, we examined RFs as predictors of class membership using the three-step procedure (R3STEP) [72], that is, a multinomial logistic regression accounting for uncertainty in class membership, which is not considered in regular or penalized regression models [e.g., 13]. Those models also included age, gender, and education level as control variables.

#### Correlational network analyses

For the network analyses, we used the R packages *bootnet* [73], *qgraph* [74], and *mgm* [75]. We calculated cross-sectional partial correlational networks of RFs using a mixed graphical model (mgm). For all models, RFs were the variables—called nodes—of interest. Interrelations between the nodes represent partial correlations and are called edges.

The mgm estimation employs a penalty approach for false-positive findings (least absolute shrinkage and selection operator method, LASSO [76]). The LASSO approach shrinks small edge weights to zero. To choose the tuning parameter, we used the Extended Bayesian Information Criterion (EBIC), setting its hyperparameter to λ = 0.25 [77], while λ = 0.50 was used for sensitivity analyses. A threshold was not employed for a more sensitive approach. We applied bootstrapping with 1000 draws to evaluate the robustness of edge-weight estimates based on 95% confidence intervals. We used correlation stability (CS) coefficients to examine centrality stability (expected correlation: 0.70) along with the corresponding stability plots. CS coefficients above 0.50 allow for a valid interpretation of centrality indices [22]. We used *strength* as centrality index that describes how well a node directly connects to other nodes (i.e., the sum of absolute edge weights).

First, we estimated pre-pandemic RF network models to examine between-RF interrelations at baseline and investigated the moderator effects of age, gender, and educational level on edge weights [78]. Second, we used LGMM to identify trajectories of psychopathological symptoms, COVID-19-related rumination and stress-related growth during the pandemic. Third, we examined pre-pandemic RFs as predictors of those trajectories. Fourth, we employed network modeling to examine the associations of pre-pandemic RFs with individual intercepts and slopes of mental health outcomes to investigate whether RFs relate to between-individual differences in overall mental health (i.e., intercepts) and between-individual differences in dynamics over time (i.e., slopes). The moderator effects of age, gender, and educational level on the links between RFs and individual intercepts and slopes were examined in sensitivity analyses.

## Results

### Sample characteristics

Our sample comprised 1275 respondents (*M*_age_ = 50.06, *SD* = 13.49, range: 20–95 years, 51.5% women). Three respondents had no school degree (0.2%), 13.1% reported to have 9 years of school, 32.1% had 10 years of school, 19.8% had an A-level exam, 31.8% held a university degree, and 3.1% completed a doctoral degree. The sample comprised a comparable proportion of women, but was significantly older than the German general population and more educated. Psychopathological symptoms were comparable to the pre-pandemic German general population (details can be found in a previous publication [42]).

### Dropout analyses

Respondents included in our study were significantly older than those who dropped out after the baseline assessment, *t*(2005) = 8.00, *p* < 0.001, *d* = 0.37, and more likely to be women, χ^2^(1) = 2.11, *p* < 0.001, *Cramer’s V* = 0.11, but were equally educated, *t*(2005) = 1.63, *p* = 0.103, *d* = 0.08. Moreover, they reported lower psychopathological symptom levels in February 2020, *t*(2004) = 2.07, *p* = 0.039, *d* = 0.10, lower levels of dispositional resilience, *t*(2004) = −4.23, *p* < 0.001, *d* = −0.20, and SOC, *t*(2004) = −3.21, *p* = 0.001, *d* = −0.15, while there were no differences in other RFs, *p* ≥ 0.079. A comparison of complete cases versus non-complete cases can be found in Supplementary Material SM5. Thus, dropout was somewhat selective, which should be considered when interpreting the following results.

### Handling of missing data

Data on RFs was complete, while data on mental health outcomes was partly missing. For psychopathological symptoms, the proportion of missing data ranged between 0.1% (February 2020) and 24.1% (November 2020), all respondents had at least three timepoints available, and 99.5% had at least four timepoints available. Missing data for COVID-19-related rumination ranged between 3.5% (March 2020) and 25% (November 2020) per assessment wave. Nine respondents (0.7%) had only two timepoints available. For stress-related growth, missing data ranged between 10.4% (April 2020) and 25.3% (November 2020). For 143 respondents (11.2%), data was only available for two timepoints. For 620 respondents (48.6%), data was complete across all timepoints and outcomes. Regression-based tests indicated that missing data was missing completely at random for 83.3% of the variables, while data for 16.7% of the variables was missing at random. Missing data was only relevant to LGMM and handled using FIML.

### Baseline resilience factor network

**Fig. 1:**
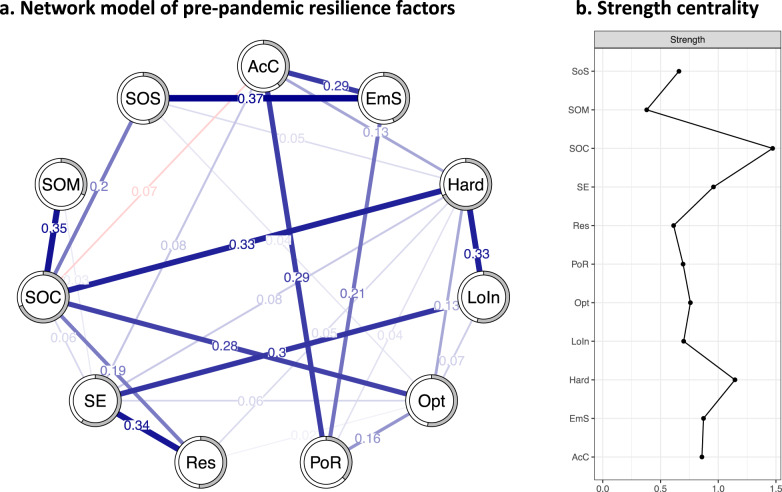
Network model of pre-pandemic resilience factors. Network of pre-pandemic resilience factors in February 2020 (**a**) and strength centrality (**b**). Absolute values of partial correlations. Blue lines indicate positive relationships, red lines negative relationships. Wider lines represent stronger associations. Predictability of nodes is indicated by the gray parts of the circles surrounding each node. Strength centrality is shown on the right. AcC = active coping; EmS = emotional support (coping); Hard = hardiness; LoIn = internal locus of control; Opt = optimism; PoR = positive reframing (coping); Res = dispositional resilience; SE = self-efficacy; SOC = sense of coherence; SOM = sense of mastery; SOS = social support.

The network model of pre-pandemic RFs is presented in Fig. 1 (bivariate correlations are presented in Supplementary Material SM6). Of 55 possible edges, 27 were included in the model, of which 26 were positive (96.3%). Bootstrapped confidence intervals of edge-weight parameters indicated acceptable precision (see Supplementary Material SM7). The strongest associations were found between (1) coping using emotional support and social support (*r* = 0.37), (2) SOC and sense of mastery (*r* = 0.35), and (3) dispositional resilience and self-efficacy (*r* = 0.34). SOC and active coping had the only negative interrelation (*r* = −0.07).

#### Centrality

The correlation stability coefficient (*r* = 0.75) allowed for a valid interpretation of centrality (see Supplementary Material SM7 for the respective plot). Strength centrality indicated that SOC was the most central node.

#### Predictability

In general, predictability was high and ranged from *R*^*2*^ = 0.32 for sense of mastery to *R*^*2*^ = 0.69 for SOC.

#### Moderation

Moderation analyses indicated that edge weights were not amplified by respondents’ age, gender, and educational level.

### Trajectories of mental health during the pandemic

**Fig. 2:**
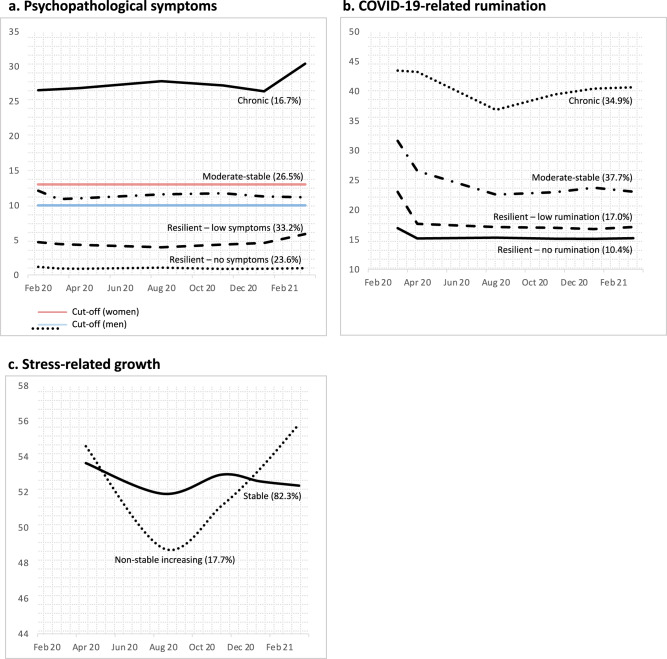
Group means of trajectories of psychopathological symptoms, COVID-19-related rumination and stress-related growth. Group means of trajectories of psychopathological symptoms (**a**), COVID-19-related rumination (**b**) and stress-related growth (**c**). Red and blue horizontal lines in panel (**a**) represent cutoff scores for the German version of the Brief Symptom Inventory [47], which were derived from a clinical sample [109], while no norm values were available for COVID-19-related rumination and stress-related growth. Percentages reflect proportions relative to the total sample of 1275 respondents.

Figure 2 shows the trajectories of psychopathological symptoms, COVID-19-related rumination, and stress-related growth during the first year of the pandemic (see Supplementary Material SM8 for descriptive data).

#### Psychopathological symptoms

We tested 1–5-class solutions for psychopathological symptoms. Consistent decreases in AIC, BIC and SSBIC and a significant BLRT test suggested a 4-class solution with linear slope (see Table 2), while the inclusion of quadratic slopes resulted in convergence problems without providing evidence for relevant quadratic effects. The final model comprised four classes, with two classes showing resilient patterns, that is, trajectories of stable good health with no-to-low psychopathological symptoms (23.6% and 33.2%, respectively). Stable symptoms at a moderate level were found for 26.5% of the sample, while 16.7% showed chronically elevated symptoms, with a later further increase in symptoms.

**Table 2:** Fit indices for latent growth mixture models (LGMM) for psychopathological symptoms, COVID-19-related rumination, and stress-related growth.

Class number	Max LL	AIC	SSBIC	Entropy	BLRT *p*	% smallest class
**Psychopathological symptoms (linear slopes)**						
1	−26879.34	53782.69	53806.38	–	–	–
2	−25994.30	52024.60	52060.13	0.76	<0.001	33.6%
3	−25776.24	61600.48	51647.86	0.73	<0.001	20.9%
4*	−25706.15	51472.31	51531.53	0.69	<0.001	16.7%
5	−25690.03	51452.06	51523.13	0.73	0.267	2.0%
**COVID-19-related rumination (linear slopes)**						
1	−23634.14	47290.27	47311.99	–	–	–
2	−23276.22	46586.44	46674.00	0.77	<0.001	47.7%
3	−23076.20	46198.40	46243.80	0.73	<0.001	12.2%
4*	−23004.74	46067.48	46124.73	0.72	<0.001	10.4%
5^a^	−22981.87	46033.75	46102.85	0.64	<0.001	10.3%
6^a^	−22968.30	46018.60	46099.54	0.61	0.118	10.3%
**Stress-related growth (quadratic and linear slopes)**						
1	−20979.81	41987.63	42015.27	–	–	–
2*	−20807.57	41663.14	41710.52	0.45	<0.001	17.7%
3	−20786.94	41641.87	41709.00	0.47	0.667	8.7%
4	−20807.57	41703.14	41790.00	0.73	1.00	<1%
5^a^	−20703.38	41514.75	41621.36	0.64	<0.001	2.0%

% smallest class = percentage of respondents in the smallest class.

*Max LL* maximized log likelihood value, *AIC* Akaike information criteria, *SSBIC* sample-size-adjusted Bayesian information criterion, *BLRT* parametric bootstrapped likelihood ratio test.

*Selected model.

^a^Problems with model convergence.

#### Rumination

For rumination, we tested 1–6-class solutions. Decreases in AIC, BIC and SSBIC suggested a 4-class solution with linear slope, while adding additional classes and including a quadratic slope resulted in convergence problems and derived no theoretically meaningful classes (e.g., separate classes with only small mean-level differences). Thus, we opted for the more parsimonious 4-class solution. Two classes showed resilient responses with initially decreasing and later stable no-to-low rumination (10.4% and 17.0%, respectively). Another 37.7% also showed initial decreases in rumination that stabilized at a moderate level. Chronically high levels of rumination were shown by 34.9% of the respondents. In contrast to other trajectories, those respondents showed an increase in rumination during the second COVID-19 wave captured by our study.

#### Stress-related growth

We examined 1–5-class solutions for stress-related growth, with studies including additional quadratic slopes showing better model fit. Decreases in AIC, SSBIC and BLRT suggested the final solution comprising two classes: most individuals (82.3%) showed relatively stable levels of stress-related growth, while a sub-population (17.7%) demonstrated a less stable pattern with initial decreases and later increases in stress-related growth.

For each model, individual-level intercepts and slopes were saved for later network analyses. This approach was further supported by significant or close-to-significant variances of intercepts and slopes in all models, except for the variance of slopes for moderate-stable trajectories of psychopathological symptoms (*p* = 0.313) and rumination (*p* = 0.462).

### Resilience factors as predictors of class membership

#### Psychopathological symptoms

Most consistently, SOC was associated with a smaller likelihood of moderate-stable and chronic trajectories compared to resilience responses, OR = 0.60–0.76 (see Table 3). Similar, but with smaller effect size, dispositional resilience was related to resilience trajectories, OR = 0.92–0.94. Higher levels of social support were associated with a smaller likelihood of chronic (but not moderate-stable) trajectories, OR = 0.87, while higher levels of sense of mastery were associated with a decreased likelihood of moderate-stable compared to resilient trajectories, OR = 0.91. Interestingly, in the combined model, including all RFs and sociodemographic characteristics, some RFs showed inverse associations with psychopathological symptoms. Active coping, coping using emotional support and positive reframing were associated with a greater likelihood of chronic compared to resilience trajectories. Higher age was associated with a higher likelihood of moderate-stable and chronic trajectories compared to resilience trajectories, OR = 1.03–1.04.

**Table 3:** Prediction of trajectory membership based on resilience factors and sociodemographic data.

Variable	OR	95% CI	*p*	OR	95% CI	*p*	OR	95% CI	*p*
**Psychopathological symptoms**									
***Resilient (no symptoms)***		***vs. Chronic***		***vs. Moderate-stable***			***vs. Resilient (low symptoms)***		
Active coping	**1.59**	[1.18, 2.14]	0.003	**1.25**	[1.01, 1.55]	0.041	1.18	[0.98, 1.42]	0.083
Coping using emotional support	**1.61**	[1.22, 2.13]	0.001	1.18	[0.85, 1.37]	0.098	1.06	[0.89, 1.26]	0.509
Dispositional resilience	**0.92**	[0.87, 0.96]	<0.001	**0.94**	[0.91, 0.98]	0.004	**0.94**	[0.91, 0.98]	0.003
Hardiness	0.97	[0.88, 1.08]	0.576	0.95	[0.88, 1.03]	0.244	0.98	[0.91, 1.05]	0.494
Locus of control	0.88	[0.62, 1.26]	0.496	0.94	[0.72, 1.25]	0.684	**0.76**	[0.59, 0.99]	0.046
Optimism	1.07	[0.68, 1.70]	0.766	0.84	[0.60, 1.17]	0.289	0.81	[0.59, 1.12]	0.201
Positive reframing	**1.40**	[1.07, 1.83]	0.003	1.19	[0.98, 1.45]	0.072	1.14	[0.98, 1.33]	0.089
Self-efficacy	1.06	[0.80, 1.41]	0.696	1.08	[0.85, 1.37]	0.523	1.17	[0.93, 1.47]	0.192
Sense of coherence	**0.60**	[0.53, 0.68]	<0.001	**0.76**	[0.70, 0.82]	<0.001	**0.89**	[0.82, 0.96]	0.004
Sense of mastery	0.98	[0.92, 1.16]	0.786	**0.91**	[0.83, 1.00]	0.038	0.96	[0.88, 1.05]	0.388
Social support	**0.87**	[0.79, 0.95]	0.003	0.94	[0.88, 1.01]	0.109	1.00	[0.93, 1.08]	0.976
Age	**1.03**	[1.00, 1.06]	0.040	**1.04**	[1.02, 1.07]	<0.001	**1.03**	[1.01, 1.05]	0.014
Gender	0.94	[0.43, 2.04]	0.874	0.74	[0.41, 1.33]	0.312	0.68	[0.39, 1.18]	0.168
Educational level	0.85	[0.60, 1.22]	0.375	1.05	[0.80, 1.39]	0.708	1.09	[0.84, 1.41]	0.526
**COVID-19-related rumination**									
***Resilient (no rumination)***		***vs. Chronic***		***vs. Moderate-stable***			***vs. Resilient (low rumination)***		
Active coping	0.86	[0.71, 1.04]	0.123	0.99	[0.83, 1.19]	0.916	0.97	[0.77, 1.23]	0.809
Coping using emotional support	**1.44**	[1.16, 1.78]	0.001	**1.26**	[1.03, 1.53]	0.023	1.24	[0.97, 1.59]	0.088
Dispositional resilience	**0.95**	[0.91, 1.00]	0.041	0.96	[0.92, 1.00]	0.052	0.97	[0.92, 1.03]	0.356
Hardiness	1.03	[0.96, 1.10]	0.441	0.99	[0.93, 1.06]	0.868	0.99	[0.91, 1.09]	0.871
Locus of control	**1.41**	[1.12, 1.77]	0.004	1.02	[0.81, 1.27]	0.883	1.20	[0.88, 1.62]	0.245
Optimism	1.24	[0.96, 1.59]	0.098	1.13	[0.89, 1.43]	0.324	1.20	[0.87, 1.64]	0.262
Positive reframing	**1.32**	[1.11, 1.56]	0.002	1.11	[0.93, 1.31]	0.245	1.24	[1.00, 1.53]	0.052
Self-efficacy	1.01	[0.85, 1.21]	0.879	1.03	[0.86, 1.23]	0.747	1.10	[0.87, 1.38]	0.432
Sense of coherence	**0.89**	[0.84, 0.94]	<0.001	0.97	[0.92, 1.02]	0.202	0.99	[0.91, 1.07]	0.733
Sense of mastery	0.97	[0.87, 1.08]	0.589	1.08	[0.97, 1.20]	0.169	1.02	[0.89, 1.17]	0.782
Social support	0.95	[0.89, 1.01]	0.097	1.03	[0.97, 1.09]	0.306	0.97	[0.91, 1.05]	0.494
Age	**1.03**	[1.01, 1.06]	0.006	**1.03**	[1.00, 1.05]	0.024	1.01	[0.98, 1.04]	0.547
Gender	0.62	[0.35, 1.11]	0.110	0.65	[0.96, 1.49]	0.131	0.80	[0.38, 1.68]	0.561
Educational level	1.11	[0.88, 1.40]	0.397	1.20	[0.96, 1.49]	0.113	1.35	[1.00, 1.84]	0.054
**Stress-related growth**									
***Stable***	***vs. Instable-increasing***								
Active coping	1.01	[0.83, 1.23]	0.921						
Coping using emotional support	1.02	[0.86, 1.21]	0.881						
Dispositional resilience	1.02	[0.99, 1.06]	0.154						
Hardiness	1.00	[0.93, 1.06]	0.908						
Locus of control	1.12	[0.86. 1.47]	0.393						
Optimism	**1.49**	[1.14, 1.94]	0.004						
Positive reframing	1.04	[0.88, 1.23]	0.670						
Self-efficacy	0.91	[0.76, 1.09]	0.308						
Sense of coherence	0.97	[0.93, 1.01]	0.111						
Sense of mastery	1.06	[0.96, 1.17]	0.271						
Social support	1.01	[0.95, 1.07]	0.816						
Age	0.98	[0.96, 1.00]	0.057						
Gender	1.31	[0.81, 2.10]	0.270						
Educational level	0.82	[0.66, 1.01]	0.058						

Results of the multinomial logistic regression analyses. Resilience trajectories (no symptoms) are used as reference class for psychopathological symptoms and COVID-19-related rumination. Stable trajectories are used as reference class for stress-related growth. Estimates take into account the uncertainty in class assignment. Significant estimates are highlighted in bold.

*OR o*dds ratio.

#### Rumination

Again, higher levels of SOC, OR = 0.89, and dispositional resilience, OR = 0.95, were associated with a smaller likelihood of chronic compared to resilience trajectories. As for psychopathological symptoms, coping using emotional support, OR = 1.44, and positive reframing, OR = 1.32, were related to chronic compared to resilience trajectories. Additionally, an internal locus of control was related to a higher likelihood of chronic compared to resilience trajectories, OR = 1.41. Higher age was linked to greater likelihood of chronic and moderate-stable compared to resilience trajectories, ORs = 1.03.

#### Stress-related growth

For stress-related growth, only optimism was associated with a higher likelihood of instable-increasing compared to stable trajectories, OR = 1.49. Except for close-to-significant effects of age and educational level, with higher age and higher education being associated with more stable trajectories, other variables showed no link with trajectories of stress-related growth.

### Networks of resilience factors and individual intercepts

Bivariate correlations between individual intercepts and RFs are presented in Supplementary Material SM9.

#### Psychopathological symptoms

**Fig. 3:**
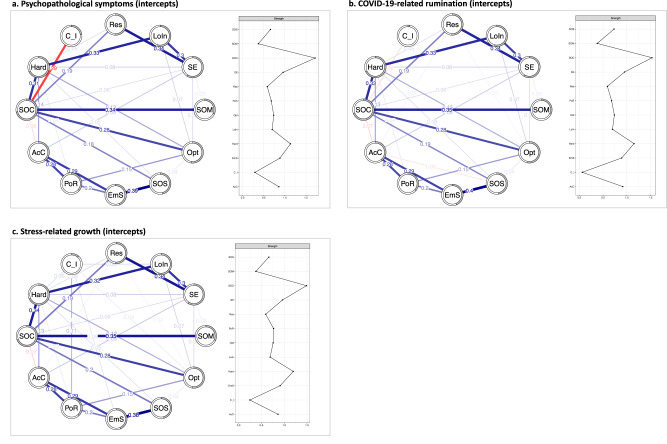
Network models of pre-pandemic resilience factors and individual intercepts for psychopathological symptoms, COVID-19-related rumination, and stress-related growth. Network model of pre-pandemic resilience factors and individual intercepts for psychopathological symptoms (**a**), COVID-19-related rumination (**b**), and stress-related growth (**c**) along with strength centrality. Absolute values of partial correlations. Blue lines indicate positive relationships, red lines negative relationships. Wider lines represent stronger associations. The predictability of nodes is indicated by the gray parts of the circles surrounding each node. AcC = active coping; C_I = individual intercepts for the respective mental health outcome; EmS = emotional support (coping); Hard = hardiness; LoIn = internal locus of control; Opt = optimism; PoR = positive reframing (coping); PS = psychopathological symptoms; Res = dispositional resilience; Rum = COVID-19-related rumination; SE = self-efficacy; SOC = sense of coherence; SOM = sense of mastery; SOS = social support.

Figure 3a shows the partial correlation network of pre-pandemic RFs and individual intercepts derived from the LGMM on psychopathological symptoms. Of 66 possible edges, 27 survived regularization, of which 25 were positive (92.6%). Bootstrapped confidence intervals of edge-weight parameters indicated sufficient precision (see Supplementary Material SM10). The strongest associations emerged between coping using emotional support and social support (*r* = 0.39) as well as between SOC and sense of mastery (*r* = 0.34), while the strongest negative link was found between SOC and individual intercepts (*r* = −0.29), that is, a higher SOC was associated with less severe symptoms and vice versa.

##### Centrality

The correlation stability coefficient (*cs* = 0.75, see Supplementary Material SM10) allowed for a valid interpretation of centrality, with SOC being the most central node. Individual intercepts showed the lowest centrality with their unique link with SOC but no associations with other RFs.

##### Predictability

The predictability for individual intercepts was moderate, with *R*^*2*^ = 22.6%.

#### COVID-19-related rumination

The network model of pre-pandemic RFs and individual intercepts derived from the LGMM on rumination are depicted in Fig. 3b. After regularization, of 66 possible edges, 28 were included in the final network. Of those, 25 were positive (89.3%). Again, bootstrapped confidence intervals of edge weights showed sufficient precision. The strongest links emerged between coping using emotional and social support (*r* = 0.40), and between SOC and sense of mastery (*r* = 0.35). Individual intercepts of COVID-19-related rumination showed only a small negative association with SOC (*r* = −0.06), with a stronger SOC being associated with lower levels of rumination and vice versa.

##### Centrality

The correlation stability coefficient (*cs* = 0.75) allowed for a valid interpretation of centrality indicators. While SOC was the most central node, individual intercepts for rumination had the smallest impact on the network.

##### Predictability

This was also evidenced by our analyses on predictability, with RFs only accounting for 2.6% of the differences in rumination intercepts.

#### Stress-related growth

Figure 3c shows the network model, including pre-pandemic RFs and individual intercepts from the LGMM on stress-related growth. After regularization, 30 edges of possible 66 edges were identified, of which 27 were positive (90%). Bootstrapped confidence intervals of edge weights supported sufficient precision. Again, the strongest links emerged between coping using emotional support and social support (*r* = 0.38) as well as between SOC and sense of mastery (*r* = 0.35), while links to individual intercepts of stress-related growth were positive but small (*r*s ≤0.11). Higher levels of stress-related growth were associated with more positive reframing (*r* = 0.11), an internal locus of control (*r* = 0.06), higher levels of hardiness (*r* = 0.06), more coping using emotional support (*r* = 0.05), and stronger optimism (*r* = 0.04).

##### Centrality

Based on a high correlation stability coefficient (*cs* = 0.75), centrality indicators can be interpreted validly. Again, SOC was the most central node, while individual intercepts for stress-related growth were least central.

##### Predictability

Together, RFs accounted for 11.4% of the variance in individual intercepts of stress-related growth.

### Networks of resilience factors and individual slopes

A different pattern of results emerged for individual slopes. Bivariate associations presented in Supplementary Material SM9 showed no relationships between RFs and individual slopes for psychopathological symptoms and COVID-19-related rumination, while small links emerged between hardiness, locus of control and self-efficacy, and slopes for stress-related growth. However, for none of the mental health outcomes, relationships between slopes and RFs survived regularization (see Supplementary Material SM11 for network models), suggesting no unique associations of RFs and dynamics over time. This was further evidenced by bootstrapped confidence intervals of edge weights, which showed low uncertainty for edges involving individual slopes (see Supplementary Material SM12).

### Sensitivity analyses

We examined whether setting the hyperparameter λ to 0.50 (compared to 0.25) changed our results and whether associations between RFs and individual intercepts and slopes were amplified by age, gender, and educational level. All analyses left our results unchanged and provided support for the robustness of our findings.

## Discussion

This 1-year prospective study examined pre-stressor resilience factors (RFs) and their association with mental health during one year of stressor exposure. Like other studies on RF networks [24, 25], we found pre-stressor RFs to be strongly interrelated (study aim 1). In line with our hypotheses and previous research [12, 16–21], sense of coherence (SOC) played the most prominent role in the RF network and as a predictor of psychopathological symptoms and COVID-19-related rumination in multinomial logistic regression analyses (study aim 2). Higher levels of SOC were associated with resilience compared to less favorable trajectories. Interestingly, when simultaneously accounting for other RFs, SOC showed no association with trajectories of stress-related growth. Multinomial logistic regression models found higher levels of optimism to be associated with instable trajectories of stress-related growth. Subsequent partial correlational network models drew a more nuanced picture by showing that SOC shared a link with individual intercepts of psychopathological symptoms and rumination, that is, individual (probably rather stable) levels of distress were related to SOC. For stress-related growth, individual intercepts were associated with a larger number of RFs (i.e., positive reframing, internal locus of control, hardiness, coping using emotional support, and optimism). However, all RFs were unrelated to slopes indicating the dynamics over time (study aim 3).

To our knowledge, this study was among the first to examine the interrelations of a broad range of RFs in an adult general population sample. In the pre-pandemic network of RFs, SOC was the most central node with unique positive partial correlations with hardiness, sense of mastery, dispositional resilience, optimism, and self-efficacy, and one small negative association with active coping. On a conceptual level, the SOC components *manageability* and *meaningfulness* may overlap with the *control* and *commitment* components of hardiness [79], with *manageability* and *control* referring to the feeling of being able to handle life challenges and *meaningfulness* and *commitment* representing the belief that these challenges are a potential source of purpose and growth [80]. Parts of the *manageability* component may also overlap with sense of mastery as the belief to be in control in different life domains [81], the *competence* dimension of dispositional resilience [82] (e.g., self-reliance, independence), the positive outcome expectancy of optimism [4], and the self-perceived capability to perform a specific behavior reflected in self-efficacy [5]. Interestingly, there was also a link between SOC and social support, while none of the items of the SOC measure [55] referred to social relationships. The negative, yet small, unique association with active coping in the network model may point to differences between the concept of active coping as a set of intentional, goal-directed efforts to minimize physical, psychological and social harm of stressor exposure [83] and the SOC component *manageability*. While active coping stresses the use of own resources to handle stress, the *manageability* component of SOC explicitly includes the reliance on external resources [80]. However, to date, evidence on overlaps between different SOC dimensions and other RFs is missing, and conclusions are limited to qualitative analyses [84]. Due to the large number of questionnaires assessed in this study, we were not able to use a longer version of the SOC scales [56], allowing for the analysis of SOC components. Moreover, many scales for the assessment of RFs—including those for SOC measurement—lack robust factorial validity [85]. Thus, future research needs to improve the psychometric assessment of SOC and other RFs.

Our study was also among the first to examine a broad set of RFs and their association with mental distress during the first year of the pandemic as a major global stressor. Multinomial logistic regression analyses supported the predictive value of SOC for distress outcomes. For both psychopathological symptoms and COVID-19-related rumination, SOC emerged as a significant predictor differentiating resilience from other trajectories, with higher levels of SOC being associated with a greater likelihood of resilient responses. Other RFs being associated with more beneficial trajectories were dispositional resilience and social support; however, effect sizes for those RFs were smaller. Findings from subsequent network modeling comprising RFs and individual intercepts of mental distress outcomes supported the importance of SOC with SOC sharing the only link with intercepts of psychopathological symptoms and rumination after regularization. However, the edge weight for rumination was much smaller in size, which was in line with the result that SOC only differentiated between resilience and chronic trajectories for rumination. Thus, our results suggest that it matters which RF is examined as predictor, with SOC being a particularly important predictor in our study.

In line with previous research [12, 16–21], our findings point to SOC’s incremental validity beyond other RFs for psychopathological symptoms and rumination and thereby challenge the notion that the association of SOC and mental health only arises from the (cross-sectional) overlap between SOC and mental health measures [86, 87]. This raises the question of what accounts for the unique value of SOC. Our network of pre-pandemic RFs suggests that SOC combines relevant aspects of other RFs (i.e., control beliefs and meaning), which may explain its associations with psychopathological symptoms and rumination. At the same time, our findings do not answer the question of what SOC adds beyond other RFs, as we did not examine SOC components and focused on SOC’s overlap with other RFs. Previous studies suggested that the SOC component *meaningfulness* may account for its incremental validity [19], however, future research using network models and a dimensional assessment of SOC is needed [18].

While findings were similar for psychopathological symptoms and rumination, analyses on stress-related growth as the only positive mental health outcome yielded different results. Latent growth modeling identified two distinct classes, with the larger class showing rather stable trajectories of stress-related growth, while another class presented both initial decreases and later increases in stress-related growth. Being a member of the latter class was more likely for those with higher levels of optimism. Our network models point to a more heterogeneous picture by showing (small) unique relationships between intercepts of stress-related growth and a larger number of RFs, that is, optimism, positive reframing, internal locus of control, hardiness, and coping using emotional support. These associations are in line with a previous meta-analysis finding posttraumatic growth to be associated with higher levels of optimism and reappraisal [88] and may reflect that optimistic reframing forms a base for stress-related growth. This notion is further supported by research into *perceived benefits* [89], which were found to be positively associated with positive reframing after stressful life events [90]. The association with locus of control may tie in with research into posttraumatic growth, suggesting that the perception of growth may also help individuals to perceive (illusionary) control [91]. Moreover, links with hardiness may derive from its *commitment* component explicitly addressing aspects of meaning [92]. Surprisingly, SOC was neither identified as a predictor of trajectories of stress-related growth in the multinomial regression analyses nor was SOC a relevant correlate of stress-related growth intercepts in the network model, although the *meaningfulness* component explicitly comprises growth from life challenges. Future studies need to examine correlates of stress-related growth in the context of multifaceted stressors.

The results of the current study also point to a sizable problem of resilience research by showing that—after regularization—a set of 11 RFs accounts for only 22.6% of the variance in intercepts of psychopathological symptoms, and RFs explained 2.6% in the variance of rumination intercepts, and 11.4% of the differences in intercepts for stress-related growth. Moreover, for none of the mental health outcomes, we found significant associations with individual dynamics over time. These findings are in line with the so-called ‘resilience paradox’ [93], describing the fact that neither single RFs nor their sum can account for the complex phenomenon of resilience. Especially for stress-related growth and rumination, associations were weak, which might reflect that many RF assessments have been optimized for high correlations with psychopathological symptoms (i.e., items have been selected to maximize the correlation with symptom measures [54, 58]). However, it is crucial to focus on transdiagnostic outcomes like rumination that are involved in the onset and persistence of multiple mental disorders [94]. Thus, future resilience research needs to broaden the scope of resilient outcomes beyond mental distress [26].

Interestingly, in combined models (i.e., in LGMM and network modeling), we found evidence for, at first sight, paradox associations. For example, active coping and coping using emotional support were associated with a higher probability of chronic compared to resilience trajectories for psychopathological symptoms. In line with a similar study [95], for rumination, an internal locus of control was associated with a greater likelihood of chronic responses. This ties in with the idea that RFs and coping strategies need to fit situational demands [3, 93]. While active coping and an internal locus of control might be helpful in many situations [6, 83], active coping might not be suitable in a situation with low individual scope of action, like the early stages of the pandemic. In those cases, a tendency toward active coping and the feeling of being in control may even cause distress. Also, the reliance on emotional support was limited by physical distancing as a component of containment measures. These findings support the idea that flexibility [93] might be key to successful adaptation and should inspire future research into underlying resilience mechanisms. Such research may also help to examine individual dynamics during stress exposure. Those were not linked to RFs in our study. On the one hand, this may suggest that RFs are correlates of (rather stable) levels of mental distress (or health) and are less relevant for coping with specific stressors; on the other hand, LGMM suggested substantial stability of mental health during stressor exposure supporting the importance of initial levels of mental health and distress. The finding of overall high stability is in line with robust but small changes in mental health during the pandemic [26, 96] but might have also limited the power of our analyses on slopes. Our analyses revealed mostly significant or close-to-significant variances of slopes, but future research with more acute stressors may shed further light on the links between RFs and temporal dynamics.

It is important to consider the limitations of the current study. First, this study was not preregistered. This has not been done as the study evolved into a larger longitudinal project with the unforeseen start of the COVID-19 pandemic in March 2020. As the pandemic had also major implications for our work as researchers, we developed this project as a fast response but deviated from our standard procedure of preregistrations. This may have biased our results, however, our aim of examining the interrelations of RFs and their predictive value for mental health outcomes has been consistently reported at all stages of this project [42, 43]. However, the results should be interpreted in light of this limitation and replicated in future preregistered studies. Second, the study is based on a diverse but nonrepresentative sample recruited via an online panel [44] and was observational in nature. Thus, mental health as assessed in this study does not exclusively reflect responses to stressor exposure but also spontaneous [97] and/or seasonal fluctuations [98], as we do not have a long-term assessment of pre-pandemic mental health in this sample to trace fluctuations independent of the pandemic. Third, models used in this study are based on (partial) correlations, hence preventing causal conclusions from being drawn. Fourth, a substantial number of respondents dropped out during the study period. We performed dropout analyses showing that study completers were equally educated but older and more likely to be women as compared to those who dropped out, which limits the generalizability of our findings. Fifth, RFs were not assessed repeatedly during the pandemic, and our results on RFs build on a single pre-pandemic assessment in February 2020. We decided against a repeated assessment of RFs that would have been too time-consuming, thus heightening the risk of larger dropout rates. Nevertheless, future studies need to examine a fully longitudinal RF network in the face of significant stress. These studies may also make use of elaborated assessments of stressor exposure that allow for the investigation of stressor reactivity [99]. Due to the dynamic course of the pandemic with varying stressors over time, we were not able to include such a measure of pandemic-related stress and disruptions in the present study. However, with our study taking place during the pandemic, we ensured that a major stressor was present at that time [26], allowing conclusions about resilience in line with the outcome-based resilience definition [1].

The current study enhances our understanding of RFs in the face of a major global stressor. We found 11 RFs to be highly interrelated, with SOC being the strongest component of a network of pre-pandemic RFs and the strongest predictor and correlate of resilient responses. Consistent with previous studies, SOC demonstrated incremental validity beyond other RFs. Patterns of associations for stress-related growth were different, pointing to the need for multidimensional outcomes in resilience research. In combined models, we found partially inverse associations for some RFs (e.g., active coping, coping using emotion support, locus of control, positive reframing), which were linked to less favorable responses. This may support the idea that the fit between RFs, coping strategies and situational demands may be key to successful adaptation. Thus, future studies need to shed light on mediating resilience mechanisms that may allow for a deeper understanding of resilience.

### Supplementary information

Supplemental Material 3

## Data Availability

More information on our analyses and exemplary code can be found in Supplementary Material SM4.

## Footnotes

1 In this study, we use the term ‘predict’ to refer to predictors (i.e., independent variables) in regression models. This does not imply a causal relationship between predictors and outcomes.

2 As it is debatable whether the COVID-19 pandemic constituted a traumatic stressor for large shares of the general population [41], we use the term ‘stress-related growth’ when referring to pandemic-related growth.
