# Supplementary material for: Interrelations of resilience factors and their incremental impact for mental health: insights from network modeling using a prospective study across seven timepoints
Source: Transl Psychiatry. 2023 Oct 23;13:328. doi: 10.1038/s41398-023-02603-2 (PMC10593776; doi:10.1038/s41398-023-02603-2)
Supplement: Supplementary file 1 — Supplemental Material 3 [file 41398_2023_2603_MOESM1_ESM.pdf]

Supplemental Material: Interrelations of resilience factors and their incremental impact for mental health: Insights from network modeling using a prospective study across seven timepoints

Authors: Sarah K. Schäfer, PhD[1,2,3], Jessica Fritz, PhD[4,5], M. Roxanne Sopp, PhD[2], Angela M. Kunzler, PhD[1,6], Lisa von Boros[1], Oliver Tüscher, MD/PhD[1,7,8], Anja S. Göritz, PhD[9], Klaus Lieb, MD[1,7] , and Tanja Michael, PhD[2]

[1] Leibniz Institute for Resilience Research, Mainz, Germany
[2] Department of Clinical Psychology and Psychotherapy, Saarland University, Saarbrücken, Germany
[3] Department of Clinical Psychology, Psychotherapy and Psychodiagnostics, Technische Universität Braunschweig, Braunschweig, Germany
[4] Department of Psychiatry, University of Cambridge, Cambridge, United Kingdom
[5] Department of Clinical Psychology, Philipps-University Marburg, Marburg, Germany
[6] Institute for Evidence in Medicine, Medical Center – University of Freiburg, Faculty of Medicine, University of Freiburg, Freiburg, Germany
[7] Department of Psychiatry and Psychotherapy, University Medical Center of Johannes Gutenberg University, Mainz, Germany
[8] Institute for Molecular Biology, Mainz
[9] Behavioral Health Technology, Augsburg University, Augsburg, Germany

Correspondence concerning this article should be addressed to Jun.-Prof. Dr. Sarah K. Schäfer, Leibniz Institute for Resilience Research, Wallstraße 7, 55122 Mainz, Email: sarah.schaefer@lir-mainz.de

Table of Contents

SM1. Details on assessment schedule ... 2

SM2. Items of the Perseverative Thinking Questionnaire adapted to COVID-19 ... 4

SM3. Items of the Posttraumatic Growth Inventory adapted to COVID-19 ... 4

SM4. Details on analyses in Mplus and R ... 6

SM5. Complete vs. incomplete cases ... 8

SM6. Descriptive statistics and edge weights in February 2020 ... 8

SM7. Bootstrapped confidence intervals of edge weight parameters and centrality stability for the pre-pandemic resilience factor network ... 9

SM8. Means and standard deviations for mental health outcomes over time ... 9

SM9. Correlations of resilience factors, individual intercepts and slopes ... 10

SM10. Bootstrapped edge weights and centrality stability for models including individual intercepts ... 11

SM11. Network models of resilience factors and individual slopes ... 12

SM12. Bootstrapped edge weights and centrality stability for models including individual slopes ... 13

Supplemental Material: Interrelations of resilience factors and their incremental impact for mental health: Insights from network modeling using a prospective study across seven timepoints

SM1. Details on assessment schedule

Assessment points for the current study were chosen to represent turning points of the pandemic in Germany (see Figure S1 for an illustration). The first COVID-19-related assessment took place in March 2020 (2020/03/16 – 2020/03/22, range Oxford Stringency Index[1]: 52.38 – 77.38), which marks the beginning of the first wave of the pandemic in Germany (with 7,274 cases at 2020/03/16 and 24,875 at 2020/03/22). First containment measures were put in place at that time (e.g., checks at German borders, closure of schools and nurseries, closure of non-essential shops, limited access to hospitals and nursing homes). The second assessment during the COVID-19 pandemic was conducted in April 2020 (2020/04/17 – 2020/04/23, Oxford Stringency Index: 77.38). During this time, infections were about to decrease, and first debates on relaxation of containment measures started[2]. During the summer months, infection rates and deaths were lower, also reflected in eased containment measures[3] (e.g., international travel was allowed, schools and nurseries as well as non-essential shops were opened, visiting bans in hospitals and nursing homes were relaxed). Starting in late spring[4], protests in the legitimacy of containment measures received increasing public interest during the summer[5]. The third COVID-19-related assessment took place at the end of August (2020/08/26 – 2020/08/31, Oxford Stringency Index: 63.1). During this time, containment measures were still debated[6] but also infection rates started to increase and caused first discussions on potential lockdown measures (e.g., closure of schools) during the winter months. The fourth COVID-19-related assessment was scheduled in November (2020/11/10 – 2020/11/17, Oxford Stringency Index: 66.67). At this time, infection rates still increased, and lockdown measures were put into place[7] (e.g., checks at German borders, closure of schools and nurseries, closure of non-essential shops, limited access to hospitals and nursing homes). Moreover, many people started worrying about gathering restrictions during Christmas holidays[8]. The next assessment at the beginning of January (2021/01/11 – 2021/01/17, Oxford Stringency Index: 86.9) was chosen to capture the situation after the Christmas holidays. Although restrictions were put in place before Christmas[9] and officials appealed to the public to avoid larger family gatherings at Christmas[10], infection rates increased resulting in prolonged lockdown measures during January 2021[11]. At the same time, vaccination rollout started in Germany[12] but was criticized as "gross failure" by Frauke Zipp, a neurologist and member of the advisory Leopoldina Academy of Sciences, due to insufficient doses of vaccine[13]. From January to March 2021, infection rates decreased. The last assessment in March

---

[1] The Oxford Stringency Index (Fuller et al., 2021; Hale et al., 2021) represents a composite index comprising nine containment policies. These include the cancellation of public events, workplace closures, school closures, gathering restrictions, border closures, internal movement restrictions, public transport closures, recommendations to stay at home, and stay-at-home-orders.
[2] https://p.dw.com/p/3bczk
[3] https://p.dw.com/p/3c0a8
[4] https://p.dw.com/p/3bOy6
[5] https://p.dw.com/p/3gMDv
[6] https://p.dw.com/p/3howt
[7] https://p.dw.com/p/3kXaz
[8] https://p.dw.com/p/3li5t; https://p.dw.com/p/3mdqK
[9] https://p.dw.com/p/3lobo
[10] https://p.dw.com/p/3mVLE
[11] https://p.dw.com/p/3nX98
[12] https://p.dw.com/p/3nF77
[13] https://p.dw.com/p/3nS9q

2021 (2021/03/16 – 2021/03/22, Oxford Stringency Index: 79.76) was a one-year follow-up of the initial first assessment wave.

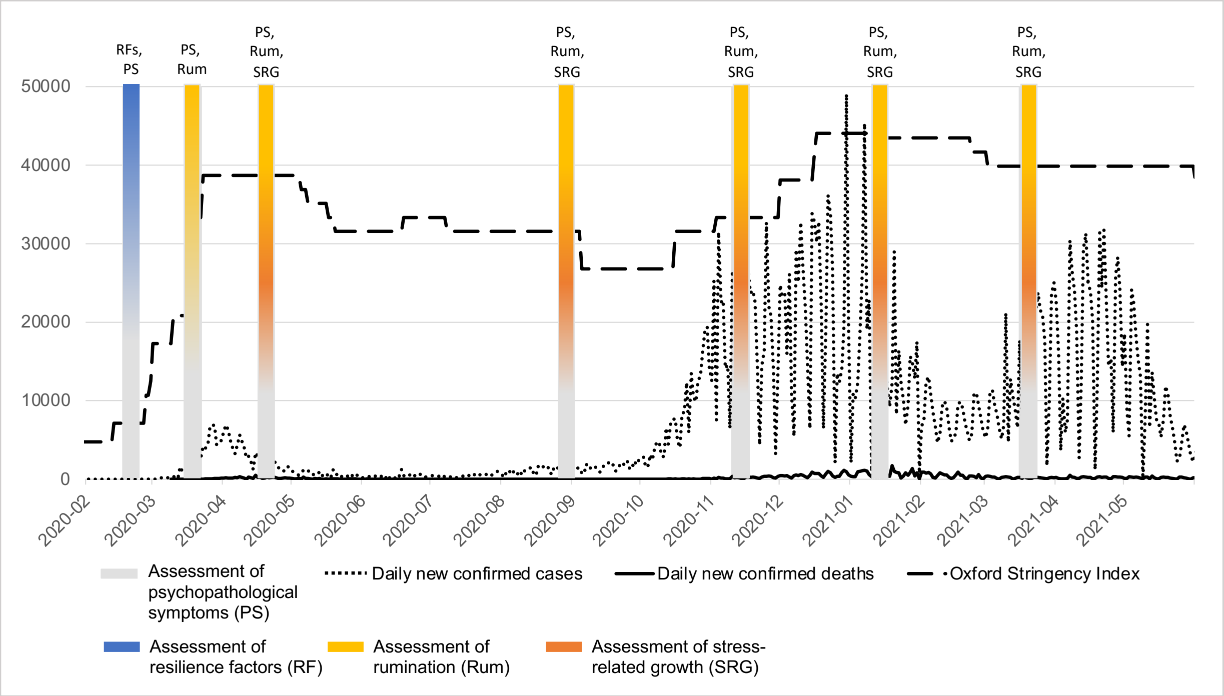

Figure S1. Schematic illustration of assessment periods, daily new cases of confirmed infections, daily new confirmed deaths associated with COVID-19 and Oxford Stringency Index
Note. The Oxford Stringency Index (Fuller et al., 2021; Hale et al., 2021) is used to quantify efforts in infection containment. RFs=Resilience factors; PS=Psychopathological symptoms, SRG=stress-related growth

SM2. Items of the Perseverative Thinking Questionnaire adapted to COVID-19 (English version)

Original publication:

Ehring, T., Zetsche, U., Weidacker, K., Wahl, K., Schönfeld, S., & Ehlers, A. (2011). The Perseverative Thinking Questionnaire (PTQ): Validation of a content-independent measure of repetitive negative thinking. *Journal of Behavior Therapy and Experimental Psychiatry*, *42*(2), 225–232.

1. The same thoughts about COVID-19 are going through my mind again and again.
2. Thoughts about COVID-19 intrude into my mind.
3. I can’t stop dwelling on thoughts about COVID-19.
4. I think about many problems connected to COVID-19 without solving any of them.
5. I can’t do anything else while thinking about COVID-19.
6. My thoughts about COVID-19 repeat themselves.
7. Thoughts about COVID-19 come to my mind without me wanting them to.
8. I get stuck on certain issues connected to COVID-19 and can’t move on.
9. I keep asking myself questions connected to COVID-19 without finding an answer.
10. My thoughts about COVID-19 prevent me from focusing on other things.
11. I keep thinking about the same issue connected to COVID-19 all the time.
12. Thoughts about COVID-19 just pop into my mind.
13. I feel driven to continue dwelling on the same issue connected to COVID-19.
14. My thoughts about COVID-19 are not much help to me.
15. My thoughts about COVID-19 take up all my attention.

All items are rated on a five-point scale: 0 = never; 1 = rarely; 2 = sometimes; 3 = often; 4 = almost always.

SM3. Items of the Posttraumatic Growth Inventory adapted to COVID-19 (English version)

Original publication:

Tedeschi, R. G. & Calhoun, L G. (1996). The posttraumatic growth inventory: Measuring the positive legacy of trauma. *Journal of Traumatic Stress, 9*, 455-471.

Due to the COVID-19 pandemic…

1. I changed my priorities about what is important in life.
2. I have a greater appreciation for the value of my own life.
3. I have developed new interests.
4. I have a greater feeling of self-reliance.
5. I have a better understanding of spiritual matters.
6. I more clearly see that I can count on people in times of trouble.
7. I established a new path for my life.
8. I have a greater sense of closeness with others.
9. I am more willing to express my emotions.
10. I know that I can handle difficulties.
11. I can do better things with my life.
12. I am better able to accept the way things work out.
13. I can better appreciate each day.
14. New opportunities are available which wouldn’t have been otherwise.

15. I have more compassion for others.
16. I put more effort into my relationships.
17. I am more likely to try to change things that need changing.
18. I have stronger religious faith.
19. I discovered that I'm stronger than I thought I was.
20. I learned a great deal about how wonderful people are.
21. I better accept needing others.

All items are rated on a five-point scale: 0 = I did not experience this; 1 = I experienced this change to a very small degree; 2 = I experienced this change to a small degree; 3 = I experienced this change to a moderate degree; 4 = I experienced this change to a great degree; 5 = I experienced this change to a very great degree.

Supplemental Material: Interrelations of resilience factors and their incremental impact for mental health: Insights from network modeling using a prospective study across seven timepoints

SM4. Details on analyses in Mplus and R

```
# Code for analyses on missing data in R version 4.4.2
library(RBtest)

# Check of missing data in mental health outcomes
df_nw <- as.data.frame(Network_data)
RBtest(df_nw)

# Code for Latent Growth Mixture Modeling in Mplus version 8.10
# Exemplary code for psychopathological symptoms, can also be used for other
mental health outcomes, for other models the number of classes needs to be
adapted from 1 to 5
# Estimation with class-specific random intercepts and slopes, which allows for
correlations of intercepts and slopes

Title: LGMM for psychopathological symptoms
  DATA: file= '/Users/XXX/df_PS.dat';
  VARIABLE: NAMES ARE CASE Age Res LoIn SE SOM Opt SOS
  EmS PoR AcC SOC Hard gen_dum edu_dum ps1 ps2 ps3 ps4 ps5 ps6 ps7;
  USEVARIABLE ARE ps1 ps2 ps3 ps4 ps5 ps6 ps7;
  Auxiliary = (R3STEP) Age Res LoIn SE SOM Opt SOS
  EmS PoR AcC SOC Hard gen_dum edu_dum
  idvariable = CASE;
  MISSING ARE all (-999); classes = c(4);
  Analysis: type = MIXTURE; estimator = MLR; STARTS = 11 9 39 100;
  STITERATIONS = 10; LRTSTARTS = 0 0 500 200;

  MODEL: %OVERALL%
  i s| y1@0 y2@1 y3@2 y4@6.5 y5@9 y6@11 y7@13;

   %c#1%
     [i s];
     i s;
     i with s;

   %c#2%
     [i s];
     i s;
     i with s;

   %c#3%
     [i s];
     i s;
     i with s;

   %c#4%
     [i s];
     i s;
     i with s;

  Output: sampstat standardized tech1
  TECH4 TECH8 TECH11 TECH14;
  Savedata:
  save=cprob;
  SAVE IS fscores;
  file=Data_classes_PS.csv;
```

```
# Code for network modeling in R version 4.4.2

# Load packages
library(bootnet)
library(dplyr)
library(mgm)
library(qgraph)
library(Hmisc)

# Load data (example run for psychopathological symptoms)
df_PS <- as.data.frame(Data_final_PS)

# Drop columns from dataframe not relevant for network models
df_PS_n <- df_PS[ , !names(df_PS) %in%
                  c("CASE", "age", "edu", "gen")]

# Calculate bivariate correlations and examine their significance
cor_PS <- rcorr(as.matrix(df_PS_n))
cor_PS

# Exemplary network model – psychopathological symptoms (intercept model)
# Network model using mgm and EBIC for lambda selection
PS_nw <- mgm(data = df_PS_n, type = rep("g", 12), level = rep(1, 12), lambdaSel
= "EBIC", lambdaGam = .25, threshold = "none") # use lamdaGam = .50 here for
sensitivity analyses

# Calculate predictability and extract predictability per node
pred_PS <- predict(PS_nw, df_PS_n, error.continuous = 'R2')
pred_PS$error

# Plot network including predictability in colorblind version
qgraph(PS_nw$pairwise$wadj,layout = 'circle',
edge.color=PS_nw$pairwise$edgecolor_cb, labels = colnames(df_PS_n),edge.labels
= TRUE, edge.label.cex=1, pie = pred_PS$error$R2, pieColor = rep('grey'),
vsize=9, esize=8,  title="a. Psychopathological symptoms - intercept model")

# Examine network stability in mgm
# stab_PS_nw <- resample(object = PS_nw, data = df_PS_n, nB = 1000)
# stab_PS_nw

# Calculate the respective network model in bootnet
PS_nw_b <- estimateNetwork(data = df_PS_n, type = rep("g", 12), level = rep(1,
12), default = "mgm", criterion = "EBIC", tuning = .25, labels = TRUE,
threshold = “none”)
summary(PS_nw_b)

# Plot for comparison
plot(PS_nw_b, edge.labels=TRUE, labels = colnames(df_PS_n), layout = 'circle')

# Centrality plot
centralityPlot(PS_nw_b, labels = colnames(df_PS_n))

# Calculate centrality stability
nw_PS_s <- bootnet(data = df_PS_n, default = "mgm", criterion = "EBIC", tuning
= .25, nBoots = 1000, nCore = 8, type = "case", threshold = “none”)
plot(nw_PS_s)

# Calculate edge weight stability
nw_PS_ew <-  bootnet(data = df_PS_n, default = "mgm", criterion = "EBIC",
tuning = .25, nBoots = 1000, nCore = 8, threshold = “none”)
plot(nw_PS_ew, order = "sample")
```

```
# Sensitivity analyses: Examine moderator effects of age, gender and educational level

# Drop column CASE from dataframe
df_PS_mod <- df_PS[ , !names(df_PS) %in%
                    c("CASE")]

# Moderator analyses – treating gender and educational level as categorical variables
PS_nw_mod <- mgm(data = df_PS_mod, type = c("g", "g", "c", "g", "c", "g", "g", "g", "g", "g", "g", "g", "g", "g"), level = c(1, 1, 6, 1, 2, 1, 1, 1, 1, 1, 1, 1, 1, 1), moderators = c(2,3,5), lambdaSel = "EBIC", lambdaGam = .25, threshold = "none")
PS_nw_mod$interactions$indicator
```

SM5. Complete vs. incomplete cases

Completers were significantly older than non-completers, $t(1273) = -2.27$, $p = .023$, $d = -0.13$, but did not differ in gender, $\chi^2(1) = 0.62$, $p = .430$, *Cramer's V* = .02, or educational level, $t(1273) = 0.67$, $p = .504$, $d = 0.40$. Completers showed lower levels of psychopathological symptoms, $t(1272) = 2.17$, $p = .031$, $d = 0.12$, and higher levels of trait resilience, $t(1273) = -3.08$, $p = .002$, $d = -0.17$, and SOC, $t(1273) = -2.07$, $p = .039$, $d = -0.12$.

SM6. Descriptive statistics and edge weights in February 2020

Table S1. Descriptive statistics and bivariate Pearson correlations of resilience factors in February 2020

	M(*SD*)	1.	2.	3.	4.	5.	6.	7.	8.	9.	10.	11.
1. Active coping	5.15 (1.69)	–										
2. Coping using emotional support	4.82 (1.87)	.48	–									
3. Hardiness	41.98 (7.01)	.39	.29	–								
4. Internal locus of control	7.61 (1.76)	.28	.16	.68	–							
5. Optimism	5.04 (1.50)	.27	.23	.63	.53	–						
6. Positive reframing	11.77 (2.32)	.27	.46	.38	.26	.39	–					
7. Self-efficacy	11.39 (3.24)	.51	.16	.63	.65	.54	.28	–				
8. Sense of coherence	45.86 (10.02)	.33	.23	.72	.56	.66	.28	.60	–			
9. Sense of mastery	11.39 (3.24)	.22	.11	.41	.36	.40	.13	.39	.57	–		
10. Social support	22.89 (5.95)	.11	.50	.51	.42	.45	.31	.41	.54	.32		
11. Dispositional resilience	70.14 (14.58)	.26	.20	.59	.53	.52	.30	.66	.62	.38	.42	–

Note. Bivariate Person correlations (all significant at $p<.001$) are displayed below the diagonal.

Supplemental Material: Interrelations of resilience factors and their incremental impact for mental health: Insights from network modeling using a prospective study across seven timepoints

SM7. Bootstrapped confidence intervals of edge weight parameters and centrality stability for the pre-pandemic resilience factor network

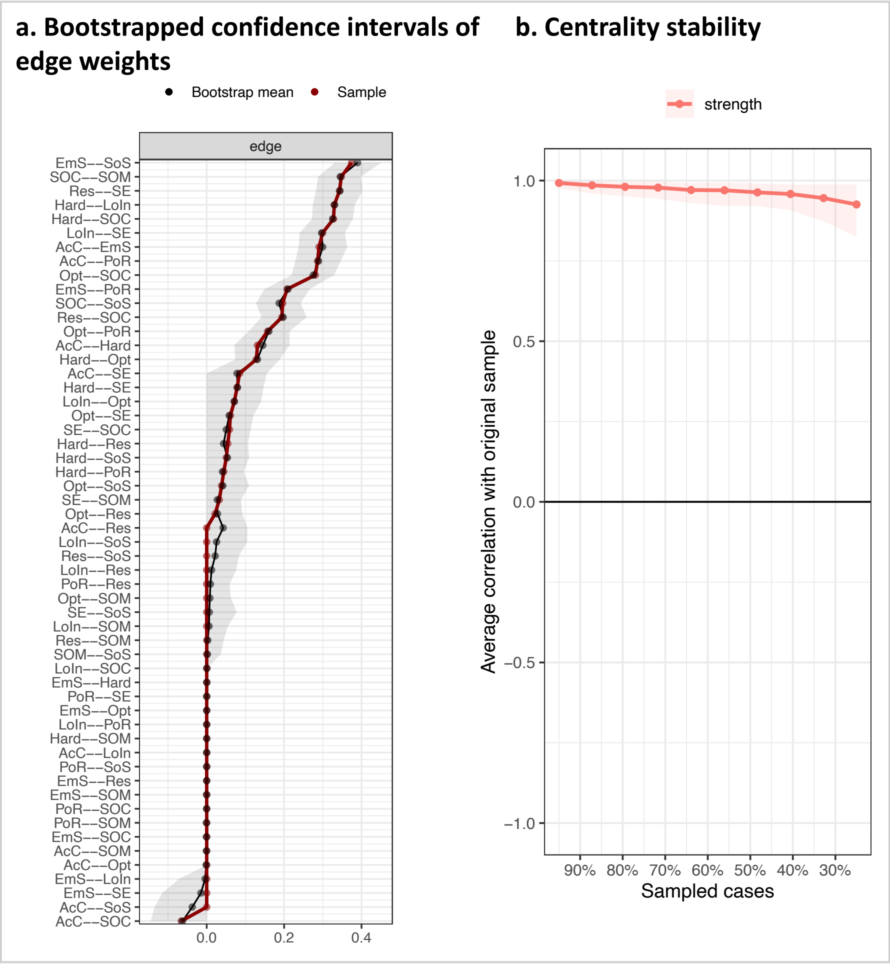

Figure S2. Bootstrapped confidence intervals of edge weights (a.) and centrality stability (b.) for the pre-pandemic resilience factor network assessed in February 2020

SM8. Means and standard deviations for mental health outcomes over time

Table S2. Means and SDs of mental health outcomes over time

	Feb 20	Mar 20	Apr 20	Aug 20	Nov 20	Jan 21	Mar 21
Psychopathological symptoms	27.43 (11.13)	27.22 (11.48)	27.19 (11.47)	26.98 (11.57)	27.28 (11.50)	26.94 (11.11)	27.53 (12.26)
COVID-19-related rumination	–	32.65 (13.44)	29.65 (13.42)	25.69 (11.81)	26.64 (12.38)	27.55 (12.67)	27.23 (12.90)
Stress-related growth	–	–	53.80 (18.96)	51.36 (18.72)	51.84 (18.75)	52.73 (18.89)	53.01 (19.07)

SM9. Correlations of resilience factors, individual intercepts and slopes

Table S3. Bivariate Pearson correlations of resilience factors, intercepts and slopes for all mental health outcomes

	1.	2.	3.	4.	5.	6.	7.	8.	9.	10.	11.	12.	13.	14.	15.	16.	.17
1. Active coping	–																
2. Coping using emotional support	.48**	–															
3. Hardiness	.39**	.29**	–														
4. Internal locus of control	.28**	.16**	.68**	–													
5. Optimism	.27**	.23**	.63**	.53**	–												
6. Positive reframing	.27**	.46**	.38**	.26**	.39**	–											
7. Self-efficacy	.51**	.16**	.63**	.65**	.54**	.28**	–										
8. Sense of coherence	.33**	.23**	.72**	.56**	.66**	.28**	.60**	–									
9. Sense of mastery	.22**	.11**	.41**	.36**	.40**	.13**	.39**	.57**	–								
10. Social support	.11**	.50**	.51**	.42**	.45**	.31**	.41**	.54**	.32**	–							
11. Dispositional resilience	.26**	.20**	.59**	.53**	.52**	.30**	.66**	.62**	.38**	.42**	–						
12. Psychopathological symptoms (intercepts)	-.02	-.02	-.34**	-.29**	-.27**	-.02	-.29**	-.47**	-.28**	-.27**	-.31**	–					
13. Psychopathological symptoms (slopes)	.00	-.01	-.01	.03	-.03	-.03	-.01	-.05	-.05	-.02	-.01	-.58**	–				
14. Rumination (intercepts)	.01	.02	.04	.02	.02	-.01	.03	.01	-.02	.01	.01	.02	-.05	–			
15. Rumination (slopes)	-.04	.01	.00	.03	.00	-.03	.04	-.01	.01	.05	.02.	-.01	.04	.00	–		
16. Stress-related growth (intercepts)	.14**	.19**	.23**	.23**	.21**	.26**	.13**	.11**	-.01	.15**	.12**	.03	.06*	.03	.01	–	
17. Stress-related growth (slopes linear)	.00	.02	-.06*	-.06*	-.03	-.04	-.06*	-.02	.00	.02	-.03	.00	-.01	.00	-.03	-.53**	–
18. Stress-related growth (slopes quadratic)	.00	-.02	.07*	.07*	.04	.05	.08*	.03	.02	.00	.03	.00	.01	.01	.04	.47**	-.68*

Note. * $p < .05$
** $p < .01$

SM10. Bootstrapped edge weights and centrality stability for models including individual intercepts

a. Resilience factors and individual intercepts for psychopathological symptoms

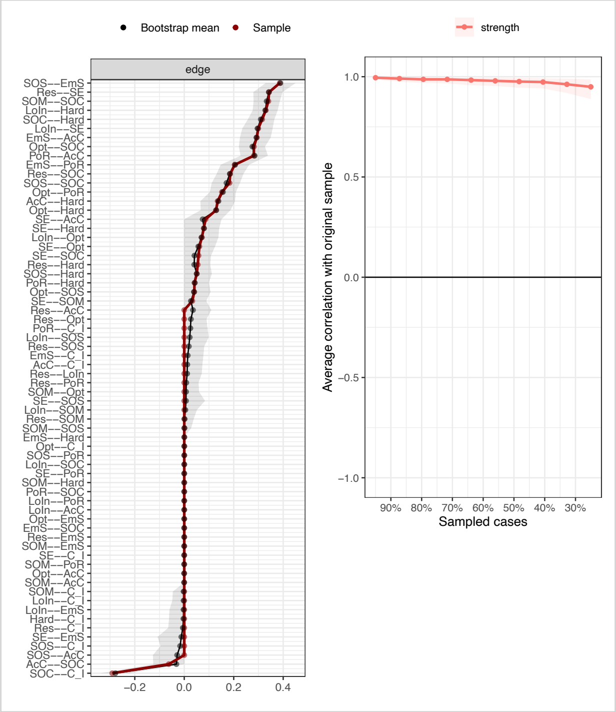

b. Resilience factors and individual intercepts for COVID-19-related rumination

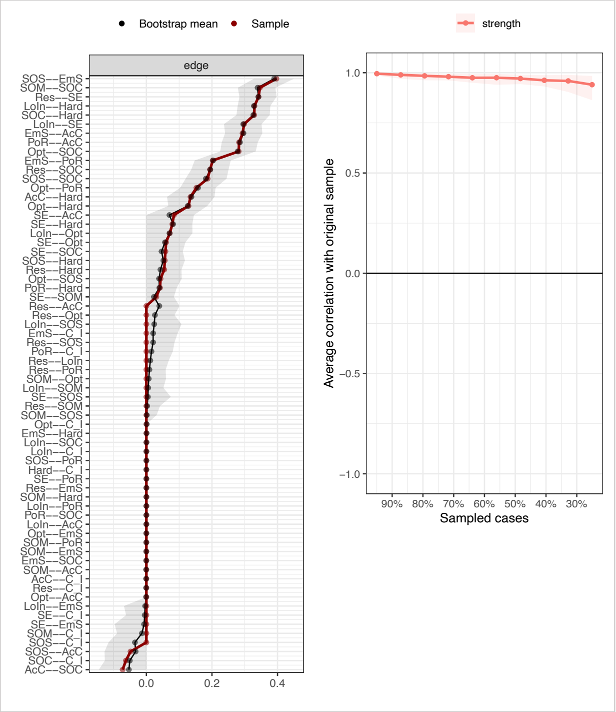

c. Resilience factors and individual intercepts for stress-related growth

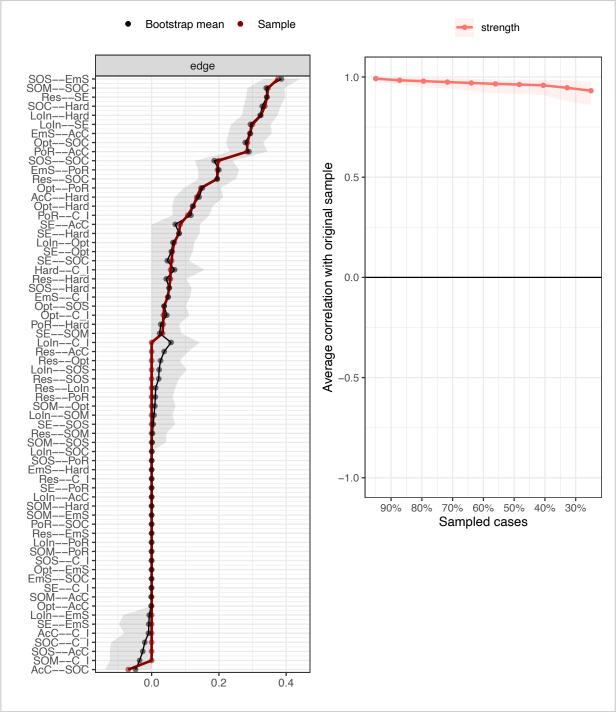

Figure S3. Bootstrapped confidence intervals of edge weights and centrality stability for the pre-pandemic resilience factor network assessed in February 2020 and individual intercepts derived from the latent growth mixture modeling (for networks see Figure 3 in the main document).

SM11. Network models of resilience factors and individual slopes

a. Psychopathological symptoms (linear slope)

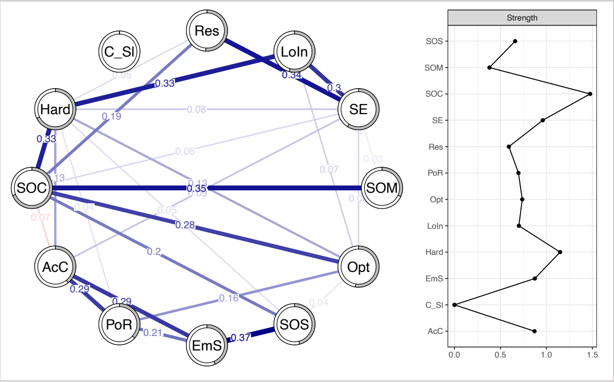

b. COVID-19-related rumination (linear slope)

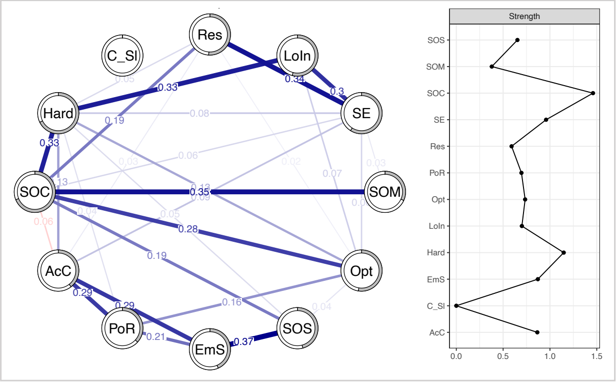

c. Stress-related growth (linear slope)

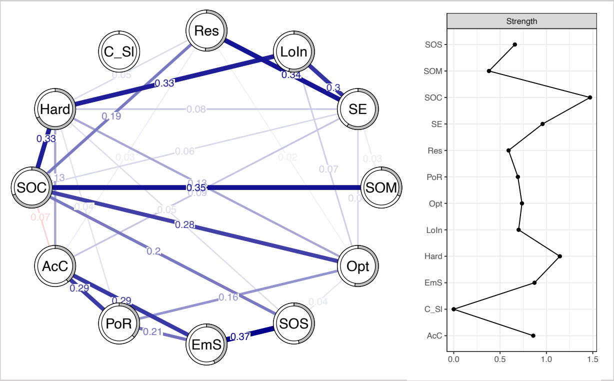

d. Stress-related growth (quadratic slope)

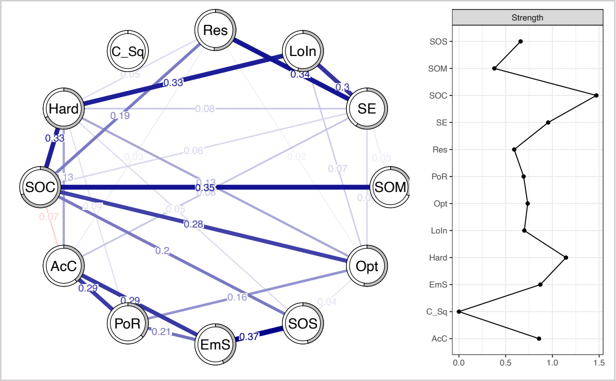

Figure S4. Network model of pre-pandemic resilience factors and individual slopes for psychopathological symptoms (a.), COVID-19-related rumination (b.), and stress-related growth (c. and d.)

Note. Absolute values of partial correlations. Blue lines indicate positive relationships, red lines negative relationships. Wider lines represent stronger associations. Predictability of nodes is indicated by the grey parts of the circles surrounding each node. AcC=Active Coping; C_Sl=Individual linear slopes for the respective mental health outcome; C_Sq=Individual quadratic slopes for the respective mental health outcome; EmS=Emotional Support (Coping); Hard=Hardiness; LoIn=Internal Locus of Control; Opt=Optimism; PoR=Positive Reframing (Coping); PS=Psychopathological Symptoms; Res=Dispositional Resilience; Rum=COVID-19-Related Rumination; SE=Self-Efficacy; SOC=Sense of Coherence; SOM=Sense of Mastery; SOS=Social Support.

Supplemental Material: Interrelations of resilience factors and their incremental impact for mental health: Insights from network modeling using a prospective study across seven timepoints

SM12. Bootstrapped edge weights and centrality stability for models including individual slopes

a. Resilience factors and individual linear slopes for psychopathological symptoms

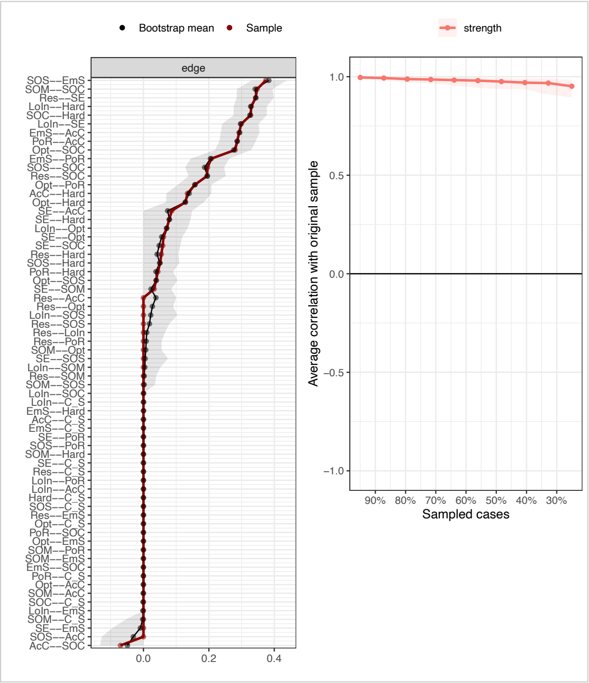

b. Resilience factors and individual linear slopes for COVID-19-related rumination

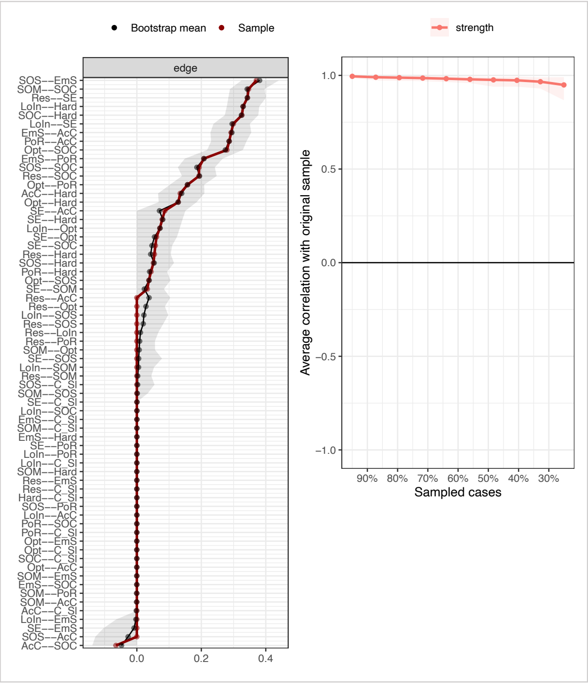

c. Resilience factors and individual linear slopes for stress-related growth

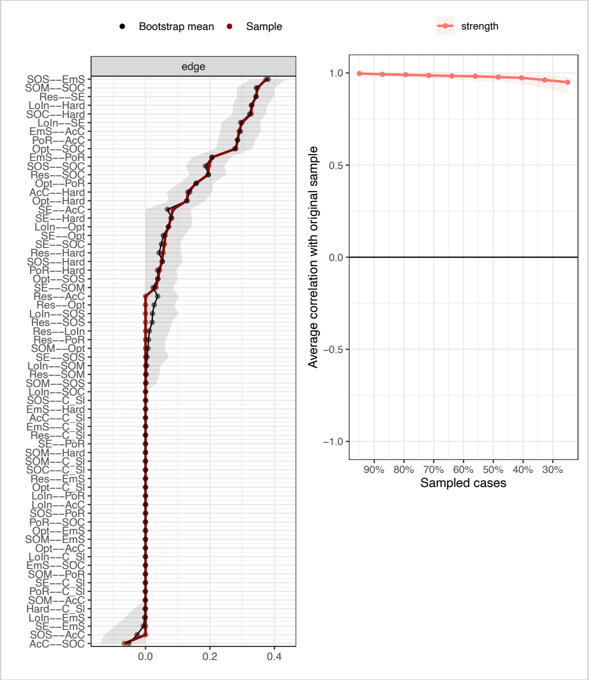

d. Resilience factors and individual quadratic slopes for stress-related growth

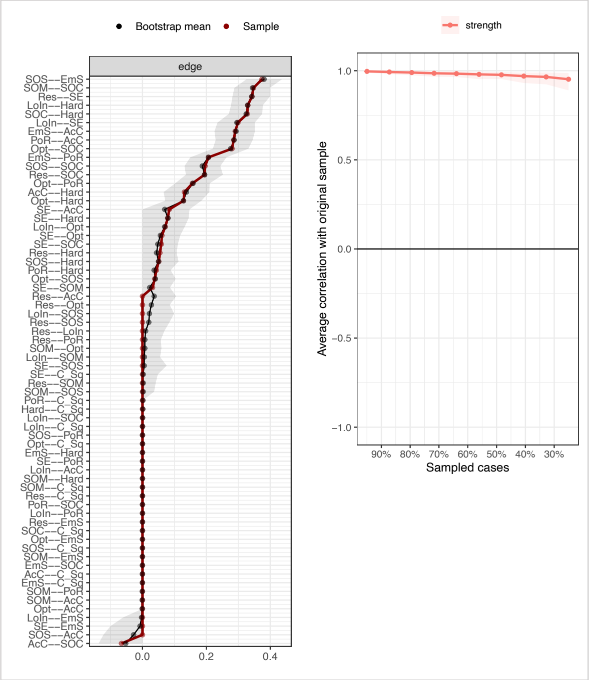

Figure S5. Bootstrapped confidence intervals of edge weights and centrality stability for the pre-pandemic resilience factor network assessed in February 2020 and individual slopes derived from the latent growth mixture modeling (for networks see Figure S4 in the main document).
